# iTRAQ-based proteomics reveals potential markers and treatment pathways for acute Achilles tendon rupture

**DOI:** 10.1186/s13018-023-04346-8

**Published:** 2023-11-09

**Authors:** Bayixiati Qianman, Aikeremu Wupuer, Tuomilisi Jiasharete, Biao Luo, Meihua Nihemaiti, Jiasharete Jielile

**Affiliations:** 1 Department of Osteopathy and Orthopedics (Ankle) Surgery, The Sixth Teaching Hospital of Xinjiang Medical University, No. 39 Wuxing South Road, Ürümqi, 830001 Xinjiang Uygur Autonomous Region China; 2 Urumqi No.1 Middle School, No. 195, West 2nd Lane, Kanas Hubei Road, Urumqi Economic and Technological Development Zone (Toutunhe District), Ürümqi, 830015 Xinjiang Uygur Autonomous Region China; 3 Department of Orthopaedics, Pingmei Shenma Medical Group General Hospital, No.1 South of Kuanggoong Middle Road, Xinhua District, Pingdingshan, 467000 Henan China; 4 Altay Traditional Chinese Medicine Hospital (Regional Kazakh Medical Hospital) in Xinjiang Uygur Autonomous Region, No. 40 Yingbin Road, Altay City, 836500 Xinjiang Uygur Autonomous Region China

**Keywords:** Acute Achilles tendon rupture, Proteomics, iTRAQ, Protein–protein interaction network, Biomarker

## Abstract

**Background:**

Due to its limited blood supply and irregular mechanical loading, the Achilles tendon is the most frequently ruptured tendon. Despite the rising incidence of acute Achilles tendon rupture (AATR), the optimal treatment remains controversial. Missed diagnoses and delayed treatments lead to poor outcomes and limited treatment options. This study aimed to identify potential biomarkers for diagnosing and developing therapies for AATR.

**Methods:**

We employed the coupled isobaric tag for relative and absolute quantitation-liquid chromatography–electrospray ionization-tandem mass spectrometry approach to investigate protein expression in tissues from AATR patients. Gene Ontology (GO) and Kyoto Encyclopedia of Genes and Genomes (KEGG) enrichment analyses were conducted to identify differentially expressed proteins (DEPs) between AATR patients and healthy individuals. A protein–protein interaction (PPI) network of DEPs was constructed using the Search Tool for the Retrieval of Interacting Genes. The screened hub genes were selectively verified by immunohistochemical staining.

**Results:**

We identified 410 DEPs between AATR patients and controls. The DEPs were significantly enriched in GO terms such as the extracellular region, extracellular region part, and defense response, as well as KEGG pathways, including complement and coagulation cascades, focal adhesion, and regulation of actin cytoskeleton. The main hub nodes in the PPI network comprised fibronectin 1 (FN1), major histocompatibility complex, class I, B (HLA-B), filamin A (FLNA), heat shock 27-kDa protein 1 (HSPB1), heat shock protein family A member 5 (HSPA5), apolipoprotein A4 (APOA4), and myosin IC (MYO1C). Although APOA4 and collagens I, II, and III were detectable in healthy tendons, immunohistochemical staining confirmed higher expression of these proteins in the acutely ruptured Achilles tendon.

**Conclusions:**

Our findings lay a foundation for further molecular studies of AATR. Inflammation and age-related degeneration may contribute to the pathogenesis of AATR. Moreover, the identified DEPs could be potential biomarkers for AATR diagnosis and treatment.

**Supplementary Information:**

The online version contains supplementary material available at 10.1186/s13018-023-04346-8.

## Background

The Achilles tendon is the strongest and largest tendon connecting the calf muscles to the heel bone, allowing us to stand on our tiptoes while walking, running, or jumping. Despite its strength, the Achilles tendon is also the most common tendon to experience rupture [1]. The inherent features, function, and limited blood supply of the Achilles tendon predispose it to both acute and chronic rupture and age-related attrition [2]. Achilles tendon rupture, which usually occurs about two inches above the heel bone, can be partial or complete. A complete rupture may be identified by a “pop” sound, followed by pain, swelling of the lower leg, and impaired movement [3]. Epidemiological studies on Achilles tendon rupture indicated a higher incidence of Achilles tendon rupture among individuals engaging in athletic activities [4]. The rising incidence of acute Achilles tendon rupture (AATR) can be attributed to the increased participation in high-demand activities by the middle-aged population.

AATR can be diagnosed based on clinical criteria [5], while ultrasound and magnetic resonance imaging (MRI) are commonly used for confirming the initial diagnosis and preoperative planning [6, 7]. However, initial evaluations may fail to identify AATR in up to 20% of cases [7]. Patients with a missed diagnosis or delayed treatment may continue to walk on the injured leg, leading to tendon gap formation and calf muscle atrophy [6], which worsens the outcomes and further limits treatment options [8]. Therefore, prompt diagnosis followed by individualized treatment planning is important for AATR patients. Despite the increasing incidence of AATR in recent decades, considerable controversy persists regarding the optimal treatment. Debate continues regarding nonoperative versus operative treatment [9–12], minimally invasive versus traditional open repair [13, 14], and early functional rehabilitation versus traditional immobilization for postoperative management [15–18].

Proteomics tools facilitate high-throughput studies of protein expression and thereby are often used to gain insight into disease mechanisms, identifying useful biomarkers and therapeutic targets [11, 15, 16, 19–21]. The isobaric tag for relative and absolute quantitation (iTRAQ)-based quantitative proteomic approach has been widely used as a powerful and sensitive proteomics tool for concurrently quantifying protein expression in up to eight different samples within a single experiment [21].

In the current study, we investigated the protein expression profile of the Achilles tendon in AATR patients using a combined iTRAQ-liquid chromatography–electrospray ionization-tandem mass spectrometry (LC–ESI–MS/MS) approach. We identified differentially expressed proteins (DEPs) between the ruptured tendons of AATR patients and the intact tendons of healthy individuals. Our findings offer insights into the pathways and biological processes involved in tendon rupture and highlight potential biomarkers for diagnosing AATR.

## Methods

### Ethics statement

Participants in the patient and control groups were willing and able to sign an informed consent form approved by the Ethics Committee of Xinjiang Medical University in China. All procedures performed in studies involving human participants were in accordance with the ethical standards of the institutional and/or national research committee and with the 1964 Helsinki Declaration and its later amendments or comparable ethical standards.

### Patient recruitment

Patients with AATR (*n* = 15, aged 20–45 years) and healthy control individuals were recruited in the First Teaching Hospital of Xinjiang Medical University, China, from 2015 to 2017. For this study, participants were randomly divided into six groups (three groups of AATR patients and three groups of healthy individuals, with five in each group). AATR diagnosis was based on the results of physical examination with a positive Thompson test in the tendon [22–24]. Complete tears of the Achilles tendon were confirmed by ultrasound [25], and histopathology analysis was performed on specimens collected from each patient.

The inclusion criteria for this study were: 1. age between 20 and 45 years; 2. gender: male; 3. complete rupture of the Achilles tendon; 4. treatment with surgery within 24 h of injury; 5. AATR occurred in a sports-related injury; 6. AATR occurred approximately 3.5–5 cm above the heel bone without calcaneal avulsion fracture (Tendon’s insertion into the calcaneus, TIC); and 7. provision of written informed consent. The exclusion criteria for this study were: 1. manifestation with spontaneous rupture of the Achilles tendon (not sports-related injury); 2. history of chronic tendonitis or prior treatment with intratendinous steroid injection; 3. prior treatment with fluoroquinolone antibiotics; 4. unwillingness or inability to sign the written informed consent form; 5. management of wounds began more than 10 h after injury; 6. prior clinical history of diabetes mellitus, hypertension, anemia, leukemia, liver or kidney dysfunction, psychosis, plantaris tendon deletion, tendon tendinopathy, or tendon calcification; and 9. calcaneal avulsion fractures. The inclusion criteria for the control group were: 1. wounds with an exposed tendon or prior amputation for severe injuries, and the management began within 6 h of injury; 2. fasting from liquids and solids for 8 h before sample collection; 3. provision of written informed consent; and 4. Achilles tendon samples were collected at the same site of the tendon. The exclusion criteria for the control group were: 1. manifestation with spontaneous rupture of the Achilles tendon; 2. history of chronic tendonitis or prior treatment with intratendinous steroid injection; 3. prior treatment with fluoroquinolone antibiotics; 4. unwillingness or inability to sign the written informed consent form; 5. wounds with exposed tendon but the management began more than 6 h after injury or treatment with amputation for the progression of diabetes or other diseases; and 6. prior clinical history of diabetes mellitus, hypertension, anemia, leukemia, liver or kidney dysfunction, psychosis, plantaris tendon deletion, tendon tendinopathy, or tendon calcification.

### Sample collection

Achilles tendon tissue samples from 15 cases were obtained from biopsies removed during surgery for rupture or open wounds (six cases) and from small tissue samples (dimension of 2 × 2 × 2 mm) removed during a needle biopsy (nine cases). Three samples were removed with one needle insertion for a biopsy (Disposable Cutting Biopsy Needle, H.S. Hospital Service Spa, Aprilia LT, Italy). Among the six male patients who underwent surgery (average age, 29.33 ± 3.83 years; range, 25–36 years), AATR involved the left limb in four patients and the right limb in two patients.

### Protein extraction, digestion, and quantification

The proteins were extracted from Achilles tendon tissue samples using the Partial Mammalian Proteome Extraction Kit (Calbiochem, La Jolla, CA, USA) and quantified using a BCA protein assay kit (Thermo Fisher Scientific, Waltham, MA, USA). Protein digestion was processed according to the filter-aided sample preparation (FASP) procedure [26] using a 30 k Microcon centrifugal filter (Millipore, Billerica, MA, USA). For each sample, 200 μg of proteins were diluted in 100-mM dithiothreitol and incubated at 95 °C for 5 min. Each sample was then cooled to room temperature (RT) and loaded into a filtration device containing 200-μL UA buffer (8-M urea, 150-mM Tris–HCl, pH 8.0) followed by centrifugation at 14,000* g* for 15 min. After centrifugation, the concentrate was mixed with 100 μL of 50-mM iodoacetamide in UA buffer and incubated in darkness at RT for 30 min followed by centrifugation at 14,000* g* for 10 min. Then, the concentrate was washed twice with 100-μL UA buffer and centrifuged at 14,000* g* for 10 min after each washing step. Next, 100 μL of 40-mM NaHCO_3_ solution was added to the concentrate, followed by centrifugation at 14,000* g* for 10 min for two times. The resulting concentrate was diluted to 40 μL with 40-mM NaHCO_3_ solution, and 2 μg of trypsin was added. After incubation overnight at 37 °C, the filter unit was transferred to a new collection tube and centrifuged at 14,000* g* for 10 min. The resulting peptides were collected and desalted on C18 cartridges (Empore™ SPE Cartridges C18 [standard density], Sigma, Eagan, MN, USA). The concentration of peptides was analyzed at optical density (OD)_280_ as described previously [26].

### iTRAQ labeling and strong cation exchange (SCX)

iTRAQ labeling was performed using an 8-plex iTRAQ labeling kit (AB Sciex, Foster City, CA, USA) according to the manufacturer’s protocol. The iTRAQ labeling was performed as follows: 3 Nt samples were, respectively, labeled with iTRAQ reagents 113, 115, and 115, and 3 At samples were labeled with iTRAQ reagents 116, 117, and 118. The three labeled samples in each group were pooled into one sample and dried in a vacuum centrifuge at RT. Based on the preferential labeling, reciprocal labeling of Nt and At samples was conducted to minimize the false-positive rate.

The iTRAQ-labeled peptides were subjected to SCX fractionation in an AKTA Purifier 100 (GE Healthcare, Marlborough, MA, USA) equipped with a polysulfethyl (PolyLC Inc., Columbia, MD, USA) column (4.6 mm × 100 mm, 5 μm, 200 Å). The peptides were eluted at a flow rate of 1 mL/min. The following gradient was applied to perform separation: 100% buffer A (10-mM KH_2_PO_4_ and 25% v/v ACN, pH 3.0) for 25 min, 0–10% buffer B (10-mM KH_2_PO_4_, 25% v/v ACN and 500-mM KCl, pH 3.0) for 7 min, 10–20% buffer B for 10 min, 20–45% buffer B for 5 min, 45–100% buffer B for 5 min, 100% buffer B for 8 min, and finally 100% buffer A for 15 min. The elution process was monitored by measuring the absorbance of wash fractions at 214 nm, and fractions were collected every 1 min. The collected fractions (approximately 33) were ultimately merged into 15 pools. Each merged fraction was concentrated via vacuum centrifugation and desalted on C18 cartridges (Empore™ SPE Cartridges C18 [standard density], Sigma). Each fraction was concentrated via vacuum centrifugation and suspended in 0.1% v/v trifluoroacetic acid. All samples were stored at − 80 °C until analysis by MS.

### Reverse-phase chromatography and MS

Samples were measured using a nanoflow HPLC (EASY-nLC 1000 system, Thermo Fisher Scientific, San Jose, CA, USA) coupled via a nanoelectrospray ion source (Thermo Fisher Scientific) to a Q Exactive mass spectrometer (Thermo Fisher Scientific). Purified and digested peptides were loaded onto an EASY column (2 cm × 100 μm, 5-μm C18) using an autosampler at a flow rate of 300 nL/min and separated on EASY column (100 mm × 75 μm, 3-μm C18). For all measurements, peptides were loaded in buffer A (0.1% formic acid) and eluted with a linear 110-min gradient of 0–55% of buffer B (0.1% formic acid and 84% acetonitrile), followed by an increase to 100% buffer B within 5 min and then 100% buffer B for 5 min.

The mass spectrometer was operated in positive ion mode, and target values for the full scan MS spectra were 3 × 10^6^ charges in the 300–1800 m/*z* range with a maximum injection time of 10 ms. Transient times corresponding to a resolution of 70,000 at m/z 200 were chosen. The top 10 most intense signals in the acquired MS spectra were selected for further MS/MS analysis. The isolation window was 2 m/*z,* and fragmentation of precursor ions was performed through higher energy collisional dissociation with the normalized collision energy of 30 eV. MS/MS scans were obtained at a resolution of 17,500 at m/z 200 and a maximum injection time of 60 ms. Dynamic exclusion was set to 30 s. The underfill ratio was defined as 0.1%. The raw data have been uploaded to the PRoteomics IDEntifications (PRIDE) database for open access with Accession number: PXD046451.

### Data analysis

The raw data were analyzed using Proteome Discoverer 1.4 software (Thermo Fisher Scientific). The search for the fragmentation spectra was performed using the MASCOT search engine embedded in Proteome Discoverer against the human decoy database (04-05-2016, 151,919 entries, http://www.uniprot.org). The following search parameters were applied: monoisotopic mass, trypsin as the cleavage enzyme, two missed cleavages, carbamidomethylation of cysteine, iTRAQ 8-plex of peptide N-term, and iTRAQ 8-plex of lysine as fixed modifications and the oxidation of methionine as a variable modification. The mass tolerance was set to 20 ppm for precursor ions and 0.1 Da for the fragment ions. The results were filtered based on a false discovery rate (FDR) of no more than 0.01%.

The relative quantitative analysis of the proteins in the samples based on the ratios of iTRAQ reporter ions from all unique peptides representing each protein was performed using Proteome Discoverer version 1.4. The relative peak intensities of the iTRAQ reporter ions released in each MS/MS spectrum were used, and the Nt samples were employed as the reference for calculating the iTRAQ ratios for all reporter ions. Then, the final ratios obtained from the relative protein quantifications were normalized based on the median of the protein quantification ratios. Protein ratios represent the median of the unique peptides of the protein. For statistical analysis, Student’s *t*-test was used to identify significant differences between the At and Nt groups, and the FDR corresponding to each p-value was further calculated. |Fold change (FC)|> 1.2 and *p*-value < 0.05 were set as the threshold values to determine the significance of DEPs.

### Functional annotation and enrichment analysis

The protein sequences of DEPs were aligned to the Gene Ontology (GO) and Kyoto Encyclopedia of Genes and Genomes (KEGG) databases to predict and classify their possible functions. The statistical significance of enrichment was determined using the Fisher's exact test. A value of *p* < 0.05 was used as the criteria for the identification of significantly overrepresented GO terms and KEGG pathways.

### Construction of protein–protein interaction (PPI) network

The Search Tool for the Retrieval of Interacting Genes (STRING) [27] was employed to construct a PPI network of DEPs. Subsequently, the PPI network was visualized and analyzed in Cytoscape [28]. The topological properties of protein nodes in the PPI network were analyzed using the CytoNCA plugin [29].

### Immunohistochemical staining for validation of biomarkers

Achilles tendon tissue samples from AATR patients and healthy control individuals were fixed in 4% paraformaldehyde, dehydrated in ethanol, embedded in paraffin, and sectioned at 4 μm. The sections were stained with hematoxylin and eosin (H and E) for histopathological examination. For immunohistochemical analysis, the sections were dewaxed in xylene, rehydrated in a standard graded series of ethanol, and washed in phosphate-buffered saline (PBS) three times for 5 min each time. Then, the sections were incubated with 3% H_2_O_2_ in methanol for 10 min to block the endogenous peroxidase activity. After being subjected to antigen retrieval by boiling in 0.01 M sodium citrate buffer (pH 6.0) for 15 min, sections were allowed to cool naturally. Subsequently, sections were incubated in 10% normal goat serum at RT for 30 min before incubation overnight at 4 °C with antibodies of apolipoprotein A4 (APOA4), collagen I, collagen II, or collagen III (dilution, 1:100; Abcam, Cambridge, UK). The next day, incubation with the primary antibodies was continued for 30 min at RT. Then, the sections were exposed to the secondary antibody for 30 min at RT. Next, the sections were incubated with biotin–streptavidin complex for 30 min. After washing, sections were immersed in diaminobenzidine hydrochloride (DAB) solution for 10 min. Sections were counterstained with hematoxylin for 30 s, dehydrated in ethanol, and mounted with neutral balsam.

The immunohistochemical staining intensity of each biopsy sample was evaluated independently by two pathologists using a microscope with an objective magnification of 400× . A semi-quantitative scoring system was employed, with staining intensity scored as follows: 0 for colorless, 1 for yellow, and 2 for brown. The average score from five microscopic fields was taken as the staining score for each section. Statistical analysis was performed using SPSS 21.0 (IBM Corp., Armonk, NY, USA). Differences between two groups were determined using the Chi-square test, with a *p*-value less than 0.05 considered statistically significant.

## Results

### Proteins expressed in Achilles tendon tissue as identified by iTRAQ

**Fig. 1:**
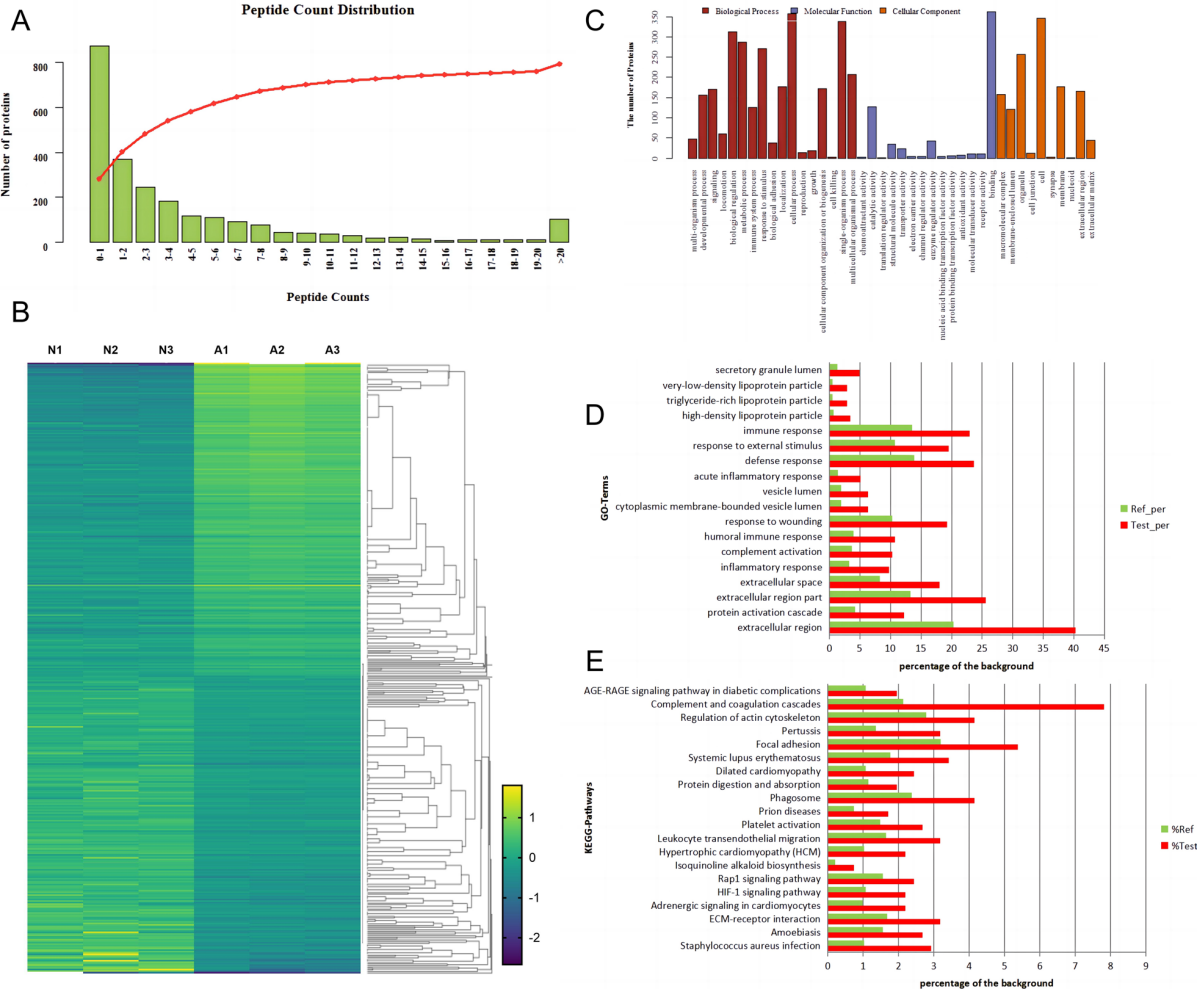
**A** Unique peptides for the identified proteins. The *x*-axis values represent the number of unique peptides, and the *y*-axis values represent the number of proteins. **B** Heatmap of significant DEPs with 210 up-regulation and 200 down-regulation in At group compared to the healthy control tendons. Colors ranging from blue to yellow correspond to low to high expression levels. **C** Functional classification of AATR-related proteins within the categories of biological process, molecular function, and cellular component. **D** Significantly overrepresented GO terms identified by enrichment analysis. The *x*-axis values represent the percentage of the background, and the *y*-axis values represent the term of enriched GO terms. **E** Significantly overrepresented KEGG pathways identified by enrichment analysis. The *x*-axis values represent the percentage of the background, and the *y*-axis values represent the term of enriched KEGG pathways

A total of 2439 proteins were identified in Achilles tendon samples (Additional file 1: Table S1 and Additional file 2: Table S2). Of those proteins, 1098 were inferred from more than two unique peptides (Fig. 1A). The molecular weights and predicted isoelectric points of the identified proteins were in the ranges of 6.04–3813.65 kDa and 3.78–12.02, respectively.

### Proteins expressed uniquely in the ruptured Achilles tendon

To identify the proteins differentially expressed following Achilles tendon rupture, Student’s *t*-test was performed to compare the expression levels of each protein between the AATR and healthy control tendon samples, and 410 DEPs were identified according to the specific criteria (FC > 1.2 and *p* < 0.05, Additional file 3: Table S3). Among these proteins, 210 were up-regulated, and 200 were down-regulated in the AATR (At) group compared with the Nt group. Hierarchical clustering showed the Nt and At samples formed tight clusters, with Nt tissues classified in the same cluster, while At tissues formed a cluster with a distinct pattern of gene expression (Fig. 1B). Compared with the Nt group, the At group showed significant up-regulation of ceruloplasmin, APOA4, complement C1r subcomponent, neutrophil defensin 1 OS, fibrinogen beta chain, immunoglobulin lambda-like polypeptide 5, integrin beta-2, insulin-like growth factor-binding protein complex acid labile subunit, plasminogen activator inhibitor 1, integrin beta-3, GPI-linked NAD(P)( +)–arginine ADP-ribosyltransferase 1, granulins, myeloperoxidase, protein canopy homolog 4, platelet receptor Gi24, leucine-rich repeat, calponin homology domain-containing protein 4, alpha-1-antichymotrypsin, and fibronectin. In addition, enoyl-CoA hydratase, mitochondrial, collagen alpha-1(IV) chain, phenylalanine–tRNA ligase beta subunit, myelin protein P0, myotilin, cysteine–tRNA ligase, cytoplasmic PDZ and LIM domain protein 4, protein CutA, transforming growth factor beta-1–induced transcript 1 protein, myoglobin, aspartate aminotransferase, cytoplasmic myosin-11, putative beta-actin-like protein 3, myosin regulatory light chain 2 (skeletal muscle isoform), and collagen alpha-1(II) chain were markedly down-regulated in At group (Additional file 3: Table S3).

### Functional annotation and enrichment analysis of DEPs

According to the GO annotations, 407 DEPs had corresponding GO term annotations. These proteins were classified into three main GO groups, namely, biological processes, molecular functions, and cellular components, and were further subclassified into 41 categories (Fig. 1C). In the group of biological processes, cellular process, single-organism process, and biological regulation were the most frequent terms. In the other two main categories, cellular components and molecular functions, the most prominent terms were binding, catalytic activity, cell, organelle, and membrane. Enrichment analysis identified 18 significantly overrepresented GO terms for DEPs (*p* < 0.05). Most DEPs were enriched in the extracellular region, extracellular region part, and defense response subcategories (Fig. 1D).

From the pathway analysis, 410 DEPs were annotated in 239 pathways (Additional file 4: Table S4), and most were enriched in 20 KEGG pathways (*p* < 0.05, Fig. 1E). The largest enrichment was 32 DEPs in the complement and coagulation cascades pathway, followed by focal adhesion, regulation of actin cytoskeleton, etc.

### PPI of DEPs

**Fig. 2:**
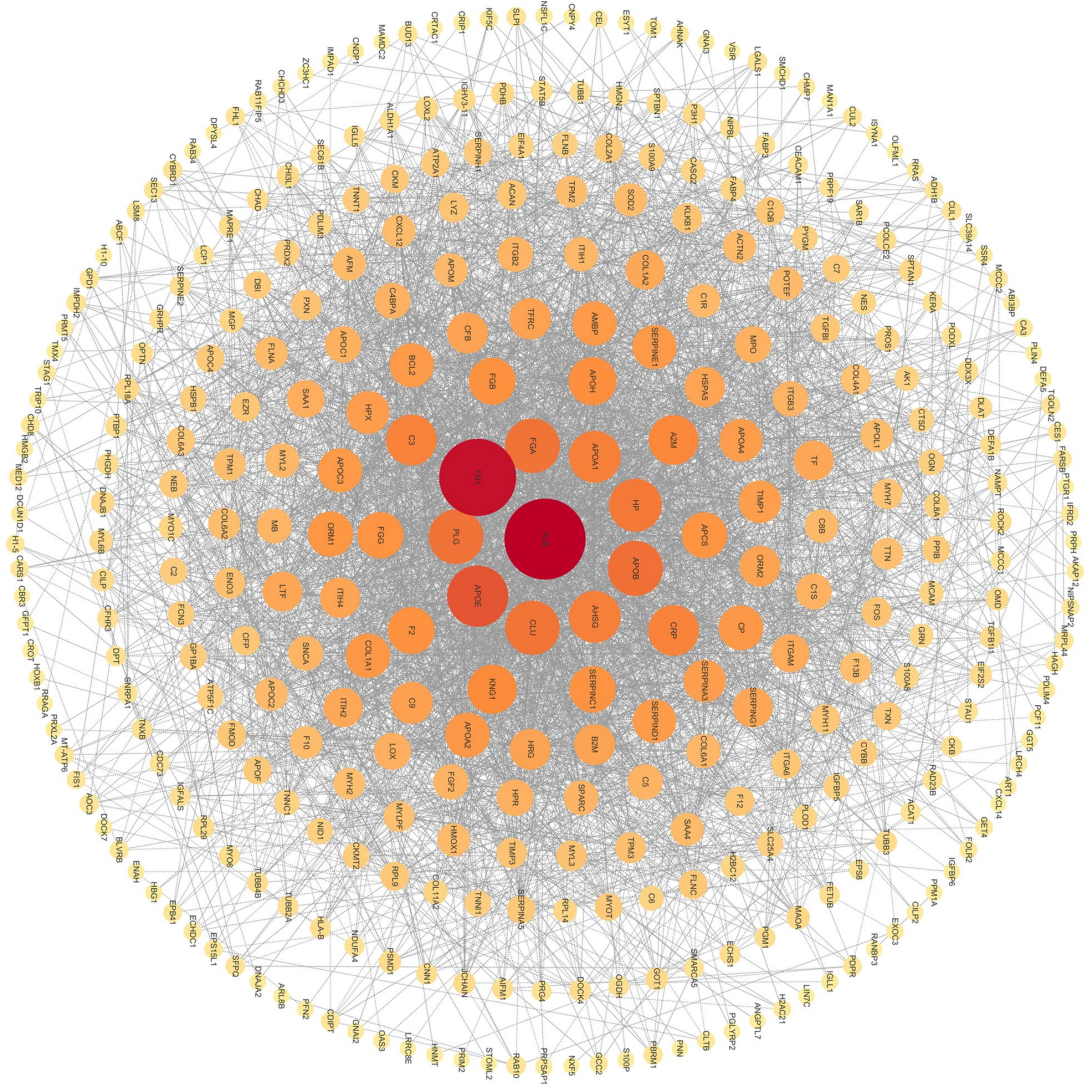
PPI network. The nodes represent proteins, and the edges represent interactions of proteins. The nodes size and red color positively correlate with the degree level of hub genes

We constructed a PPI network using STRING software to deepen our understanding of the molecular mechanisms underlying the identified DEPs and explore their potential roles in AATR. In this analysis, the identified DEPs formed a complex network containing 381 nodes and 1121 edges (Fig. 2 and Additional file 5: Table S5). Fibronectin 1 (FN1, degree = 312), major histocompatibility complex, class I, B (HLA-B, degree = 189), filamin A (FLNA, degree = 165), heat shock 27-kDa protein 1 (HSPB1, degree = 161), heat shock protein family A (Hsp70) member 5 (HSPA5, degree = 133), and myosin IC (MYO1C, degree = 109) were found to be the most important hubs in the constructed PPI network for AATR.

### Histological verification of tendon tissues from AATR patients

**Fig. 3:**
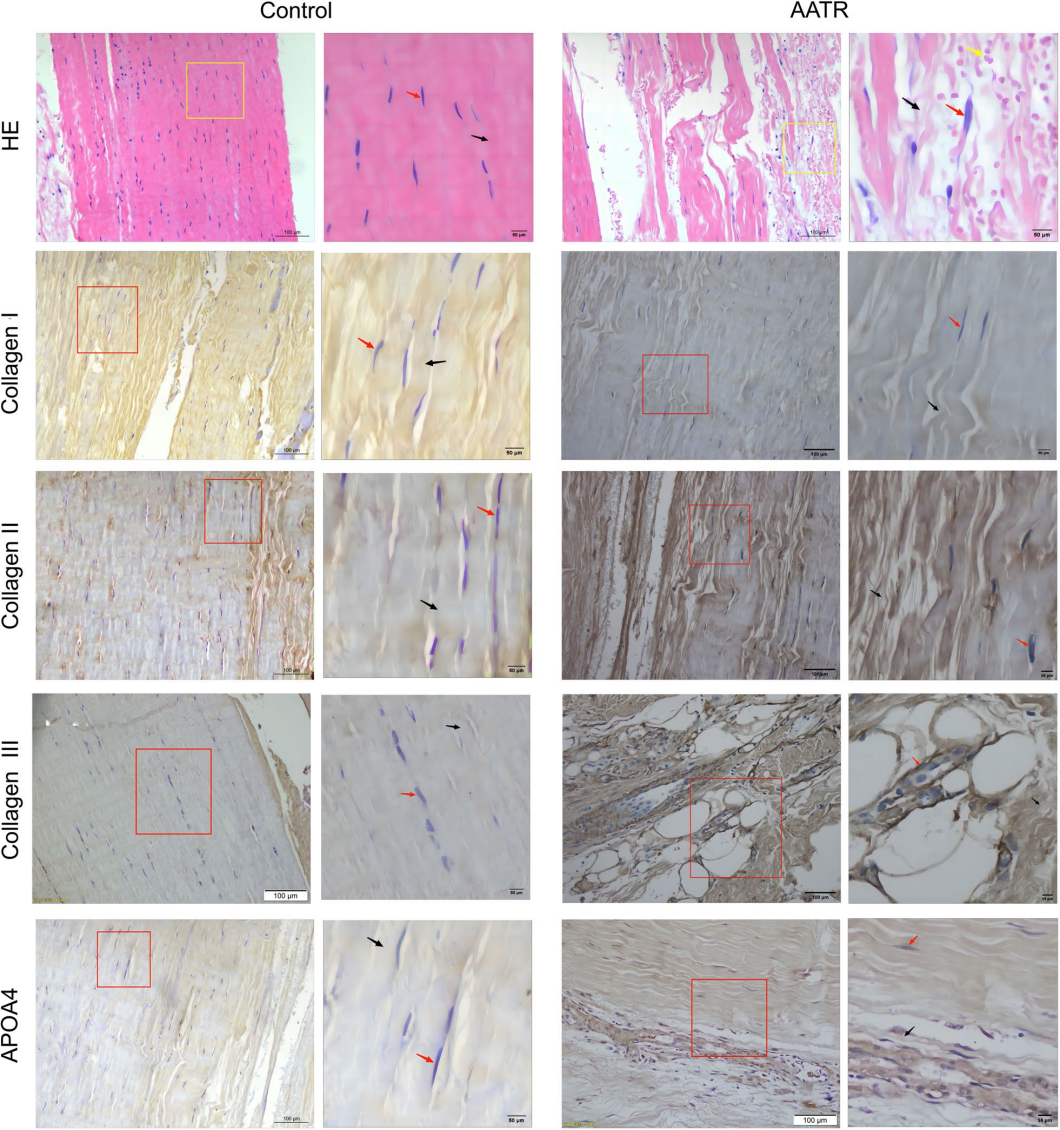
First line showing the representative H and E staining of healthy control Achilles tendon tissue and acutely ruptured Achilles tendon tissues. The enlarged scope was indicated by the yellow box. The black arrow indicates the fiber alignment. The red arrow indicated the nuclei morphology, and the yellow arrow indicated the infiltrated inflammatory cells. The second and fifth lines show the representative immunohistochemical staining for collagens I, II, and III and APOA4 in healthy control of Achilles tendon tissue and acutely ruptured Achilles tendon tissues. The enlarged scope was indicated by the red box. The red arrow indicates the nuclei morphology. The fiber plasm with staining was indicated by the black arrow

Histopathological analysis (H and E staining) was conducted on Achilles tendon tissues from AATR patients and healthy controls (Fig. 3). The healthy tendon exhibited a well-arranged cellular order and red-stained collagen fibers. Additionally, the nuclei of fibroblasts were of normal shape and centered, with no inflammatory cell infiltration. Conversely, the fibers in the AATR tendon samples were pink-stained and significantly dissociated, with a total loss of alignment. Furthermore, the nuclei of fibroblasts displayed an irregular morphology and nuclear fragmentation. Immunohistochemical staining was carried out to compare the expression levels of APOA4, collagen I, collagen II, and collagen III between the Achilles tendon tissues from AATR patients and those of healthy controls. Although expression of these four proteins was detectable in healthy tendon, these proteins were more strongly expressed in the AATR tendon samples (Fig. 3) and APOA4, which are the key DEPs identified by our analysis (Fig. 3). Semi-quantitative analysis suggested that the expression levels of APOA4, collagen I, collagen II, and collagen III were higher in AATR tendons than in healthy control ones (Table 1), aligning with our iTRAQ results. However, due to the small sample size in the present study, statistical differences between the AATR and healthy tendon groups were not significant.

**Table 1:** Semi-quantitative expression of APOA4, collagen I, collagen II, and collagen III in Achilles tendon tissues from AATR patients and healthy controls

Group	Score			*χ*^2^	*p*
	Negative	Weakly positive	Strongly positive		
*APOA4 expression*					
Control	4	7	4	3.21	
AATR	1	6	8		0.201
Total	5	13	12		
*Collagen I expression*					
Control	1	4	10		
AATR	2	7	6	2.152	0.341
Total	3	11	16		
*Collagen II expression*					
Control	2	8	5		
AATR	3	9	3	0.759	0.684
Total	5	17	8		
*Collagen III expression*					
Control	1	7	7		
AATR	2	8	5	0.733	0.693
Case	3	15	12		

Score 0, negative; score 1, weakly positive; and score 2, strongly positive. AATR, acute Achilles tendon rupture

## Discussion

In the present study, 410 DEPs in the acutely ruptured Achilles tendon were identified using an iTRAQ-based proteomic approach. GO enrichment analysis revealed that these DEPs were significantly associated with 18 GO terms, and most were enriched in the extracellular region, extracellular region part, and defense response subcategories. KEGG enrichment analysis identified 20 significantly overrepresented pathways corresponding to the DEPs, with most DEPs enriched in pathways related to the complement and coagulation cascades, focal adhesion, and regulation of actin cytoskeleton. The PPI network constructed from the DEPs selected FN1, HLA-B, FLNA, HSPB1, HSPA5, and MYO1C as hub nodes.

The prevailing consensus suggests that most tendon injuries result from cumulative wear and tear of the tendon from overuse or aging [30]. Many researchers believe that inflammation plays a role in the pathogenesis of tendinopathy, even though an acute inflammatory infiltrate is often not seen in histopathological analysis of the ruptured tendon tissue [31]. Key inflammatory mediators have consistently been reported to play crucial roles in modulating changes in the extracellular matrix within tendinopathy [32]. Consistent with the previous studies, our data showed that DEPs were significantly associated with the extracellular region, extracellular region part, and defense response GO terms. In addition, the most significantly overrepresented KEGG pathway for DEPs was the complement and coagulation cascade pathway. The complement system, as well as the coagulation system, is known to be crucially involved in the inflammatory response [33]. Fibroblasts produce the extracellular matrix components that constitute tendonous tissue [34]. The previous research demonstrated that Achilles tendon fibroblasts from old mice have reduced mobility and proliferation, a disorganized actin cytoskeleton, and altered localization of key focal adhesion proteins compared to Achilles tendon fibroblasts from young mice [35]. The AATR-related DEPs identified in the present study were consistently enriched in the focal adhesion and regulation of actin cytoskeleton pathways. These findings indicate that inflammation and age-related degeneration may work together in the pathogenesis of AATR. Understanding the interactions between inflammatory mechanisms, degeneration that occurs with aging, and Achilles tendon homeostasis would benefit the development of novel therapies for AATR.

The hub genes identified in the PPI network unraveled the potential molecular mechanism underlying the AATR pathogenesis. Fibronectin is a glycoprotein of the extracellular matrix that is involved in the process of cell adhesion and migration [36]. A systematic review revealed enhanced expression of fibronectin in tendinopathy [37]. In the current study, FN1 (P02751) was up-regulated in the tendons of AATR patients compared with those of the healthy controls and was suggested to be the top significant hub gene in our PPI network, indicating that FN1 may play a role in AATR progression. HLA-B presents foreign antigens to the immune system [38]. Hundreds of alleles of HLA-B are known, and HLA-B27 is associated with Achilles tendinitis [39]. In the current study, HLA-B15 (P30464) was up-regulated, and HLA-B41 (P30479) was down-regulated in the AATR tendons compared with the control tendons, implying that HLA-B may be involved in multiple pathways in AATR pathogenesis. FLNA (P21333) is an actin filament cross-linking protein that regulates actin cytoskeleton organization by interacting with integrins, transmembrane receptor complexes, and second messengers [40]. In the present study, FLNA was down-regulated in the AATR tendons compared with the control tendons, suggesting that the expression level of FLNA and the corresponding effects on actin cytoskeleton regulation may be involved in AATR pathogenesis. Heat shock proteins can act directly as danger signals in tendinopathy [41]. Overexpression of heat shock protein 27 is important in preventing proteolytically-induced apoptosis [42], with research showing that heat shock protein 70 inhibits apoptosis by preventing the recruitment of procaspase-9 to the Apaf-1 apoptosome [43]. In the present study, HSPB1 (P04792) was down-regulated, and HSPA5 (P11021) was up-regulated in the AATR tendon samples compared with the healthy control tendons, implying that HSPB1 and HSPA5 may be important regulators of the apoptotic process in AATR. Actin and myosin are both present in tenocytes, and myosins are actin-based molecular motors [44]. In the present study, MYO1C (O00159) was down-regulated in AATR tendon samples compared with the healthy control samples, indicating that down-regulation of MYO1C may reduce cell mobility in AATR. Altogether, the hub proteins in the PPI network were all associated with tendinopathy, and thus, they could be used as potential biomarkers for AATR diagnosis and therapy development.

Our study does have some limitations. The findings were based on the analysis of 15 AATR patients and 15 healthy individuals. Therefore, additional studies of AATR with larger sample sizes are needed to validate our findings. PPI network analysis identified the hub genes critical for the pathological progress that indicated the potential intervention targets. Further examination of these functional modules of interest could help refine the molecular pathways involved in AATR.

## Conclusions

This study employed an iTRAQ-based proteomic approach to closely examine DEPs potentially involved in the pathogenesis of AATR. Our findings enhance the understanding of the molecular mapping of AATR and underscore the role of inflammation and aging in AATR pathogenesis. The DEPs within the PPI network, including FN1, HLA-B, FLNA, HSPB1, HSPA5, and MYO1C, could potentially serve as biomarkers for AATR diagnosis and therapeutic targets.

### Supplementary Information

**Additional file 1**. **Table S1.** Details of identifed proteins.

**Additional file 2**. **Table S2.** Details of identified peptides.

**Additional file 3**. **Table S3.** Screening of differentially expressed proteins from the iTRAQ-LC/MS/MS data.

**Additional file 4**. **Table S4.** Annotation of differentially expressed proteins compared with KEGG database.

**Additional file 5**. **Table S5.** The nodes in the PPI network of differentially expressed proteins.

## Data Availability

The datasets generated and analyzed during the present study are available from the corresponding author upon reasonable request.

## Abbreviations

ATR: Achilles tendon rupture
Tp: Tendinopathy
DP: Differential proteomics
iTRAQ: Isobaric tags for relative and absolute quantitation
DTT: Dithiothreitol
GO: Gene Ontology
KEGG: Kyoto Encyclopedia of Genes and Genomes
ESI: Electrospray ionization
HPLC: High-performance liquid chromatography
APOA4: Apolipoprotein A4
ITGB2: Integrin beta-2
POTEKP: Putative beta-actin-like protein 3
MYL2: Myosin regulatory light chain 2
SERPINE1: Plasminogen activator inhibitor 1
LRCH4: Leucine-rich repeat and calponin homology domain-containing protein 4
COL2A1: Collagen alpha-1(II) chain
ITGB3: Integrin beta-3
FN1: Fibronectin
PFN1: Profilin-1
PGK1: Phosphoglycerate kinase 1
PK: Pyruvate kinase
PRDX1: Peroxiredoxin 1
